# Dengue virus infection induces myocarditis in IFNα/β receptor deficient mice

**DOI:** 10.1186/s43556-023-00150-2

**Published:** 2023-10-31

**Authors:** Chongzhi Bai, Ruoyu Wang, Qiuxia Xiong, Qian Yang, Pengcheng Han

**Affiliations:** 1 Central Laboratory, Shanxi Province Hospital of Traditional Chinese Medicine, Taiyuan, China; 2 Department of Microbiology, School of Basic Medical Sciences, Peking University Health Science Center, Beijing, China; 3 Yunnan Key Laboratory of Laboratory Medicine, Yunnan Province Clinical Research Center for Laboratory Medicine, Kunming, China; 4 School of Medicine, Zhongda Hospital, Southeast University, Nanjing, China

**Keywords:** Dengue virus (DENV), Heart, Myocarditis, Dilated cardiomyopathy, Cytokines

Dear editor:

The Dengue virus (DENV) is a positive-sense single-strained RNA virus that belongs to the Flavivirus genus within the Flaviviridae family. It is prevalent in tropical regions across more than 100 countries, causing an estimated 100–400 million infections each year. The range of diseases caused by the Dengue virus varies from mild febrile illness to fatal conditions such as Dengue Hemorrhagic Fever (DHF) or Shock Syndrome, which poses a growing threat to public health. Generally, the diseases caused by DENV are self-limiting and can resolve without intervention. However, a small proportion of patients may experience severe dengue, characterized by abnormalities in blood coagulation, plasma leakage, increased vascular fragility, and ultimately leading to DHF. In severe cases, complications such as hepatitis, neuropathy, myocarditis, or severe bleeding can occur, leading to life-threatening conditions.

A systematic investigation in dengue patients revealed that cardiac involvement occurred in 15% of those requiring hospitalization, exhibiting clinical manifestations ranging from slightly elevated cardiac biomarkers to myocarditis, pericarditis, and even death [1]. Furthermore, DENV antigens were detected in various cardiac cells, including myocardial fibers, endothelial cells, and myocardial interstitial cells, unequivocally indicating viral replication within these cell types [1, 2]. In vitro studies on the myoblast cell line H9c2 cells demonstrated the susceptibility of all four DENV serotypes [3]. These injuries could be attributed to either viral replication within specific organs or the intensified immune response during the infection [1]. The peak severity of DENV infection occurs after the virus has been eliminated by the host immune system rather than during the peak of viral load. This observation strongly implies that the host immune response plays a crucial role in the pathogenesis of DENV infection [2]. Despite the existing evidence demonstrating cardiac damage resulting from DENV infection in clinical cases and in vitro studies, there remains an urgent need to investigate the underlying mechanisms behind the development of cardiovascular diseases caused by DENV infection.

In this study, we have established a mouse model to investigate the pathogenesis of DENV in the heart. The model is based on the contemporary Dengue strain (GenBank: AF204178). We aim to evaluate the cardiac outcomes and host immune response induced by DENV infection and explore the potential mechanisms associated with these effects. To achieve this, we inoculated 4-week-old Wild-type C57BL/6 (WT) mice and IFNα/β receptor deficient (KO) mice with a dose of 10^4^ plaque-forming units (pfu) of DENV through intraperitoneal injection (IP). The uninfected group received an injection of Vero cell culture medium.

Real-time quantitative PCR (qRT-PCR) was performed to analyze DENV levels in mice's hearts and brains after 7 DPI. Elevated levels of DENV were detected in both hearts and brains of KO-infected mice, whereas no viruses were detected in WT-infected mice (Fig. 1a). We successfully established a model of DENV infection in a mammalian animal. Starting at 4 DPI, KO-infected mice exhibited reductions in body mass, but all the mice survived after the infection (Fig. S1). Masson staining was conducted on the hearts to examine the degree of pathological fibrosis. Five out of ten mice infected with DENV exhibited fibrotic hearts 10 days post-infection (DPI), whereas no fibrosis was observed in WT-infected mice. The hearts of KO-infected mice displayed significantly increased fibrotic scarring compared to KO-uninfected mice. No differences were noted in the hearts of uninfected WT and KO mice (Fig. 1b). Therefore, our study compared KO mice before and after infection. Echocardiography assessed heart function at 5, 10, and 15 DPI. Following 10 DPI, the KO-infected mice showed further left ventricle dilation, deterioration of ejection fraction and fractional shortening (Fig. 1c, d). Elevated levels of CK-MB and cTnT in KO-infected mice also indicated myocardial injury, as shown in Fig. 1e. Therefore, these findings confirm that DENV infection leads to myocardial dysfunction in KO mice.

**Fig. 1:**
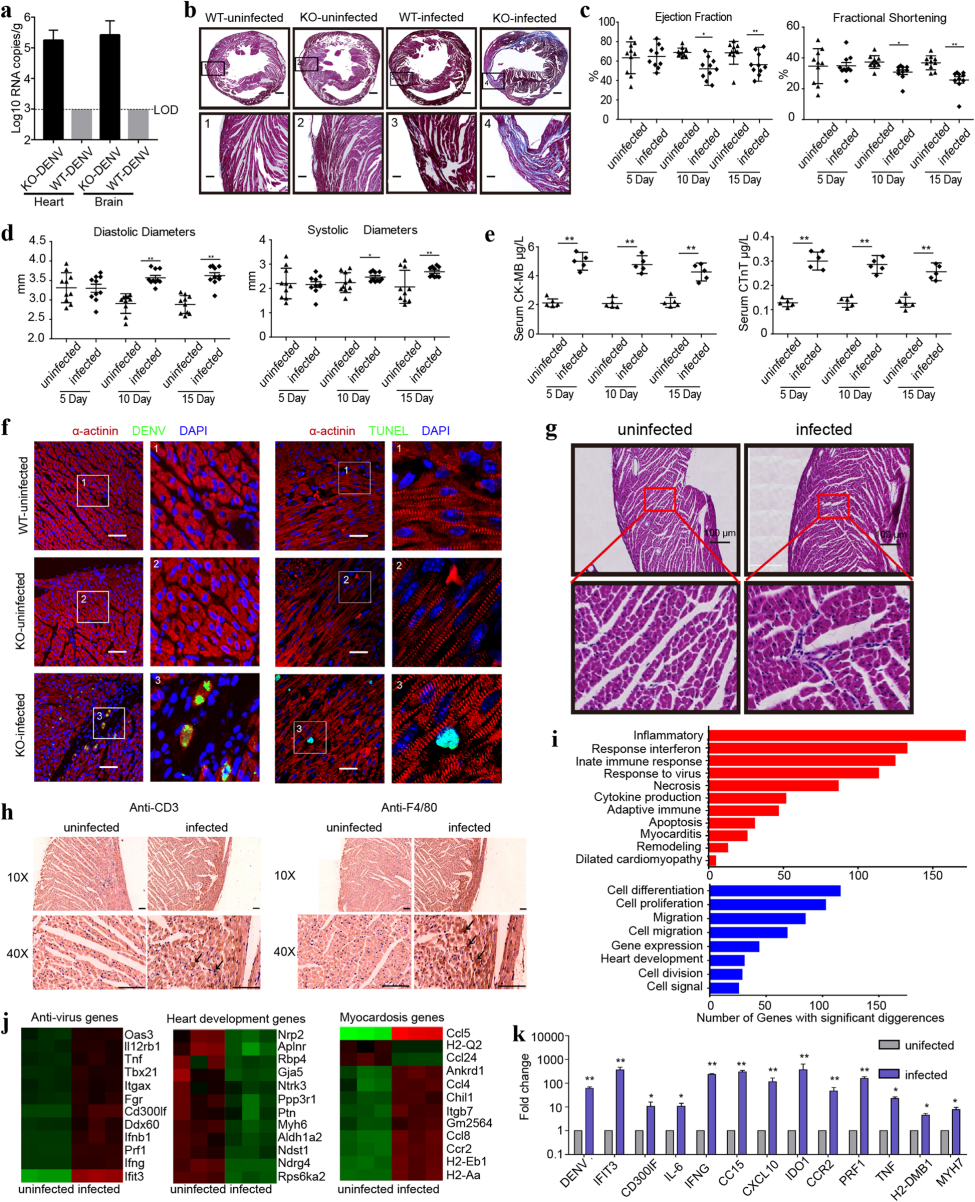
DENV infection induces myocardial immune response and myocarditis. The WT and KO groups injected with Vero cells supernatants used as control and included 5 and 10 mice, respectively. Both WT and KO mice injected with DENV contained 10 mice. **a** DENV infection was quantified in the heart and brain. **b** Masson staining of DENV-infected mice and uninfected mice. The lower panels (Scale bar, 200 μm) indicate the enlarged view in the boxed region in the upper panels (Scale bar, 500 μm). **c** Ejection fraction and fractional shortening were evaluated by echocardiography. **d** Diastolic diameters and systolic diameters were evaluated by echocardiography. **e** Serum CK-MB and cTnT level were evaluate. **f** Infection and apoptosis of cardiomyocytes in DENV-infected mouse heart. The enlarged views of the regions highlighted with the squares are shown in the right panels. Scale bar, 50 μm. **g** Heart tissue sections were subjected for HE staining. The regions highlighted with red squares in the upper panels are enlarged and displayed in the lower panels. Scale bar, 100 μm. **h** Infiltration of immune cells to KO-DENV heart was evaluated by immunohistochemistry using anti-CD3 for leukocytes or anti-F4/80 for macrophages. Scale bar, 25 μm. **i** Genes with significant differences in expression between infected and uninfected hearts were subjected to GO analyses. The most significant terms are shown for upregulated (red) and downregulated (blue) genes, respectively. **j** Heat maps show hierarchical clustering of differentially expressed genes. **k** Relative mRNA levels were determined with real-time qRT-PCR. Data are presented as mean ± SEM. *p < 0.05; **p < 0.01

To detect direct infection of mouse hearts by DENV, we employed the monoclonal antibody Z6 immunofluorescence assay. Uninfected KO mice served as the negative control. After 10 DPI, fluorescent signals indicating the presence of DENV were observed in the hearts of infected mice. We further determined that DENV positive cells were explicitly located in cardiomyocytes, as they expressed the cardiomyocyte marker α-actinin. Additionally, the TUNEL assay revealed cell apoptosis, with fluorescent signals localized in the cardiomyocyte nuclei (Fig. 1f). These results conclusively demonstrate that DENV directly infects cardiomyocytes in KO mice, leading to cardiomyocyte apoptosis.

To investigate if DENV infection of the heart leads to histopathological myocarditis, characterized by the infiltration of inflammatory cells, we performed hematoxylin–eosin (HE) staining on heart tissue samples. Our observations revealed the presence of monocytes infiltrating the hearts of infected mice (Fig. 1g). Additionally, we detected CD3^+^ leucocytes and F4/80^+^ macrophages infiltrating the heart (Fig. 1h), indicating that specific immune cell types are involved in the immune response to DENV infection in the heart. Furthermore, the flow cytometry results showed elevated number of CD3^+^CD4^+^ and NK1.1^+^ cells in the blood (Fig. S2).

RNA sequencing analysis was carried out on the hearts of uninfected and infected KO mice to identify overall gene expression changes. Examination of the differentially expressed genes revealed a significant enrichment in gene ontology associated with the inflammatory response and innate immunity. Additionally, gene networks related to cell apoptosis, necrosis, myocarditis, and dilated cardiomyopathy were observed. In contrast, various genes implicated in cell differentiation, migration, proliferation, and heart development were found to be down-regulated (Fig. 1i, j). To assess the innate immune response to DENV infection and subsequent myocarditis, qRT-PCR was employed to measure the expression levels of specific inflammatory cytokines and genes associated with myocarditis. The results demonstrated a marked increase in mRNA levels of major pro-inflammatory cytokines, such as IFIT3, IL-6, CC15, IFNγ, TNFα, and CXCL10, as well as an up-regulation of genes associated with both myocarditis and dilated cardiomyopathy, namely MYH7 and PRF1, following DENV infection (Fig. 1k).

In conclusion, our findings prove that DENV can directly infect the hearts of IFNα/β receptor knockout mice, specifically targeting cardiomyocytes within a few days following infection. The observed infiltration of inflammatory cells, myocardial fibrosis, and cardiac dysfunction indicate the development of severe myocarditis in this mouse model, which aligns with clinical evidence of cardiac dysfunction. Notably, DENV-induced myocarditis and immune response were observed exclusively in immune-deficient mice rather than wild-type mice. This suggests that individuals with compromised immunity may be particularly vulnerable to the detrimental effects of DENV infection, emphasizing the need for heightened awareness of cardiovascular symptoms within clinical settings.

In patients infected with DENV, we observed a decrease in the expression of the epigenetic modifier EZH2, caused by an increase in miR-150 and let-7e expression. EZH2 inhibition has been shown to suppress the virus through immune signaling pathways involving IL-6 and IP-10 [4]. Furthermore, NS1 induces the expression of suppressor of cytokine signaling 3 (SOCS3) driven by NF-κB and activates target cells to produce cytokines like IL-6, TNF-α, and IP-10 through TLR4/TLR2/TLR6 [5]. The cytokine storm is a pathogenic mechanism that leads to severe dengue disease and may contribute to vascular permeability [5]. Additionally, our global transcriptomes datasets will serve as valuable resources for further research into the cellular and molecular mechanisms underlying the pathological effects of DENV on heart development and cardiovascular diseases.

### Supplementary Information

**Additional file 1.**

## Data Availability

DENV strain: GenBank accession number: AF204178 (https://www.ncbi.nlm.nih.gov/nuccore/AF204178/ ). RNA-Seq data: SRA accession number: PRJNA592016.
