# Supplementary material for: Dengue virus infection induces myocarditis in IFNα/β receptor deficient mice
Source: Mol Biomed. 2023 Oct 31;4:36. doi: 10.1186/s43556-023-00150-2 (PMC10616043; doi:10.1186/s43556-023-00150-2)
Supplement: Supplementary file 1 — Additional file 1. [file 43556_2023_150_MOESM1_ESM.docx]

**Dengue Virus Infection Induces Myocarditis in**

**IFNα/β receptor deficient mice**

Chongzhi Bai ^1,2,3 #^, Ruoyu Wang ^1 #^, Qiuxia Xiong ^3 #^, Qian Yang ^1^, Pengcheng Han ^4^ *

^1^ Central Laboratory, Shanxi Province Hospital of Traditional Chinese Medicine, Taiyuan, China; ^2^ Department of Microbiology, School of Basic Medical Sciences, Peking University Health Science Center, Beijing, China; ^3^ Yunnan Key Laboratory of Laboratory Medicine, Yunnan Province Clinical Research Center for Laboratory Medicine, Kunming, China; ^4^ School of Medicine, Zhongda Hospital, Southeast University, Nanjing, China.

^*^Correspondence: 101013216@seu.edu.cn

^#^These authors contributed equally to this work.

**Keywords**

Dengue virus (DENV)；Heart；Myocarditis；Dilated cardiomyopathy; cytokines

**Supplemental material**

**Materials and Methods**

**Viruses, DENV Antibody and Cells**

DENV (GenBank accession number: AF204178) was Laboratory-stored virus. Virus stocks were propagated in mycoplasma-free Vero cells and titrated by plaque forming assay. Anti-DENV human mAb Z6 were prepared in-house. Vero cells and BHK-21 cells were maintained in DMEM, supplemented with 10% fetal bovine serum and L-glutamine at 37 ℃ with 5% CO_2_. Mosquito C6/36 cells were maintained in RPMI 1640 medium containing 10% fetal bovine serum at 28 ℃.

**Mouse Infection Experiments**

Wild-type C57BL/6 (WT) male mice were purchased from the Beijing Vital River Laboratory Animal Technology and IFNα/β receptor-deficient (KO) male mice were purchased from Institute of Laboratory Animal Science, Chinese Academy of Medical Sciences. Four-week-old male mice were inoculated by intraperitoneal injection (IP) with a dose of 10^4^ plaque-forming units (pfu) DENV or Vero cells supernatants in a volume of 50 μl. The WT and KO groups injected with Vero cells supernatants used as control and included n=5 and n=10 mice, respectively. Both WT and KO mice injected with DENV (WT-DENV and KO-DENV) contained n=10 mice. Blood through the eyeball method to detect cytokines in serum at different time points after virus infection, the mice were euthanized and the hearts were collected for histological and immunofluorescence analyses. At indicated DPI, the tissues were harvested for further analysis. The survival, weight loss, and signs of diseases were monitored for 15 days in all mice.

**Echocardiography analysis**

Heart function analysis was performed using VEVO 2100 (Visual Sonics, Toronto, Canada). Anesthesia was necessary to ensure the accurate positioning of the transducer probe and to avoid movement during the measurement. 5, 10, 15 days after infection, mice were continuously anaesthetized with 1.5–2% isoflurane (Pharmaceutical Partners of Canada, Richmond Hill, Canada) and warmed on a heated pad (37°C) for long and short-axis views of heart function was obtained in B-Mode and M-Mode. Left ventricular fractional shortening (FS), ejection fraction (EF), end-systolic dimension (LVESD) and end-diastolic dimension (LVEDD) of the left ventricular were analyzed for each mouse. All data were averaged by repeating five times per mouse.

**Immunochemistry, Histology, Tunel and Immunofluorescence**

For Histology, tissue was harvested and fixed overnight in 10% formalin solution, and 5-μm-thick heart sections were processed for histology by the hematoxylin and eosin HE and Masson staining.

For Tunnel, tissue was harvested and fixed overnight in 10% formalin solution, and 5-μm-thick heart sections were stained by using the terminal deoxynucleotidyl transferase (TdT) dioxyuridine triphosphate (dUTP) nick-end labeling assay (TUNEL) in accordance with manufacturer instructions (In Situ Cell Death Detection Kit POD; Roche, Mannheim, Germany) to visualize cell apoptosis.

For immunofluorescence, the frozen tissue sections (6 mm) were incubated with mouse primary monoclonal antibody anti-DENV (Z6; obtained in our laboratory) at 4 ℃ overnight. After washing with TBST, secondary antibody for Z6 (ab6854; Abcam), was applied for 1 hr at 37 ℃. Subsequently, the sections were washed with TBST and counterstained for DAPI (ThermoFisher Scientific).

For immunohistochemistry, the paraffin blocks of tissues were sectioned at 5 mm. Deparaffinization, rehydration and antigen retrieval were performed as described previously. The tissue sections were treated with 3% H_2_O_2_ in PBS (pH 7.6) for 10 min and blocked with normal goat serum for 10 min. The sections were then incubated at 4 ℃ overnight with primary monoclonal antibody anti-DENV (Z6; obtained in our laboratory) (1:200) (Key Resources Table). After rinsing with PBS, the primary antibodies were subsequently detected by incubation with biotinylated secondary antibody (Key Resources Table) followed by avidin-biotin-peroxidase (Vector Laboratories, USA). Specific binding was visualized using 3, 3’-Diaminobenzidine tetrahydrochloride. Sections were slightly counterstained with Mayer’s hematoxylin. Heart tissues from KO mice that were either uninfected or infected with DENV were taken for microscopic analyses.

**RNA extraction and real-time PCR analysis**

Total RNAs were extracted from cardiac tissue with RNeasy Mini Kit (tissues) (QIAGEN) and detected by real-time qRT-PCR on an ABI 7500 Fast Instrument (Applied Biosystems, US) using One Step PrimeScript RT-PCR kit (TaKaRa, Japan) according to the manufacturer’s protocol. The expression levels of genes were normalized on a log10 scale as viral RNA copies per g or per mL after comparison with GAPDH. Real-time PCR experiments were performed from three independent RNA preparations.

**ECLA**

The serum was collected after centrifugation at 800×g (Beckman Coulter, C0650) for 10 min. The CK-MB and cTnT were measured using ECLA kits in accordance with manufacturer’s instructions.

**Flow cytometry**

Flow cytometry was performed on a Cytomics™ FC 500 ([Beckman Coulter](https://www.baidu.com/link?url=kiz0tUh1lhu9S8ZnAAdwamBIpKV8S6nImUjVUranak6S3ULtiKDeiZTjB6Kf-kxe&wd=&eqid=dedb900d000054f1000000025a33458b), USA). At least 10,000 cells were evaluated. Antibodies: NK1.1 (17-5941-82; Invitrogen), CD4 (17-0042-82; Invitrogen), CD45 (12-0451-83; Invitrogen), CD3 (11-0032-82; Invitrogen).

**RNA-Seq Analysis**

Whole hearts of KO mice 10 days after DENV or Vero cells supernatants infection (three for each group) were used for global transcriptome analysis by Annoroad Co. Significantly differentially expressed genes were identified when we compared Normalized Reads Count between DENV and Vero cells supernatants infection groups with p < 0.05 and Log2FoldChange > 0.263. Significance of Gene Ontology term enrichment was estimated with Fisher’s Exact Test (p value).

**Statistics**

All data were analysed with the SPSS16.0 software for Windows. The values are presented as the mean ± SEM. P-values < 0.05 were considered significant differences. Calculations were performed in Prism 6 (Graph-Pad Software).

Table 1. Key resources table

| REAGENT or RESOURCE | SOURCE | IDENTIFIER |
| --- | --- | --- |
| Antibodies |  |  |
| Z6 | Lab production (Unpublished data) | N/A, materials available on request from lead contact |
| Rabbit Anti-CD3 antibody | Proteintech | 17617-1-AP; AB_1939430 |
| Rabbit Anti-F4/80 antibody | Bioss | bs-11182R |
| SP Kit(Rabbit) | Bioss | SP-0023 |
| [Goat Anti-Human IgG H&L (FITC)](https://www.abcam.cn/goat-human-igg-hl-alkaline-phosphatase-ab97162.html) | Abcam | ab6854; AB_955300 |
| Anti-Sarcomeric Alpha Actinin | Abcam | ab9465; AB_2155972 |
| Rabbit two-step assay kit | ZSGB-BIO | PV-9001 |
| CD45 Monoclonal Antibody, PE | Invitrogen | 12-0451-83; AB_465669 |
| CD3 Monoclonal Antibody, FITC | Invitrogen | 11-0032-82; AB_2572431 |
| CD4 Monoclonal Antibody, APC | Invitrogen | 17-0042-82; AB_469323 |
| NK1.1 Monoclonal Antibody, APC | Invitrogen | 17-5941-82; AB_469479 |
| Critical Commercial Assays |  |  |
| TUNEL kit | Roche | Cat NO: 11684817910 |
| Viral RNA Mini kits | Qiagen | Cat NO: 52906 |
| RNeasy Mini Kit | Qiagen | Cat NO: 74106 |
| One-Step PrimeScript RT-PCR Kit | Takara | Cat NO: RR064B |
| The MB isoenzyme of creatine kinas, CK-MB | Roche | Cat NO: 11821598322 |
| Elecsys Troponin T, cTnT | Roche | Cat NO: 04491815190 |
| Experimental Models: Cell Lines |  |  |
| African green monkey: Vero | ATCC | CCL-81 |
| Baby Hamster: BHK-21 | ATCC | CCL-10 |
| Mosquito: C6/36 | ATCC | CRL-1660 |
| Human: 293T | ATCC | CRL-1573 |
| Experimental Models: Organisms/Strains |  |  |
| Virus: Dengue (GenBank: AF204178) | Laboratory-stored virus | N/A, materials available on request from  lead contact |
| Mouse: C57BL/6 | Beijing Vital River Laboratory Animal Technology | 213 |
| Mouse: *Ifnar1^-/-^* | Institute of Laboratory Animal Science, Chinese Academy of Medical Sciences | 31-11-001-C-002494 |
| **Software and Algorithms** |  |  |
| SPSS12.0.1 Package | SPSS | www.brothersoft.com/spss-268827.html |
| GraphPad Prism 5.0 | GraphPad Software | www.graphpad.com/scientific-software/prism/ |

Table 2. The list of primers used in the qRT-PCR assay.

| Target genes | Primers | 5’----3’ |
| --- | --- | --- |
| Cd300lf | Cd300lf-F | GTGATGGTGATGGTCGTGGTGTG |
|  | Cd300lf-R | TCTGCTACCGCTATCCTCCAAGTG |
| Cxcl10 | Cxcl10 -F | CCAGCCGTGGTCACATCAGC |
|  | Cxcl10 -R | GCGTCGCACCTCCACATAGC |
| H2-DMb1 | H2-DMb1-F | GCAACAAGGAGAAGACGGCTCAG |
|  | H2-DMb1-R | GCTGAACCACGCAGGTGTAGAC |
| Ifit3 | Ifit3-F | GGCACCATGAACCTGAGGACAAC |
|  | Ifit3-R | ATAAGCAGCACTCCACAGCACATC |
| Myh7 | Myh7-F | GCAAGACGGTGACTGTGAAGGAG |
|  | Myh7-R | GGTTGACGGTGACGCAGAAGAG |
| IL_6 | IL-6 -F | AGGAGTGGCTAAGGACCAAGACC |
|  | IL-6 -R | CTGACCACAGTGAGGAATGTCCAC |
| Fpr2 | Fpr2-F | CCGCTGCATTTGTGTTCTGC |
|  | Fpr2-R | AAATCCAGGGCCCAACAACC |
| Tnf | Tnf-F | GCGACGTGGAACTGGCAGAAG |
|  | Tnf-R | GCCACAAGCAGGAATGAGAAGAGG |
| DENV | DENV-F | TAGAGAGCAGATCTCTGATGAA |
|  | DENV-R | TGAGAATCTCTTCGCCAAC |
| Ccl5 | Ccl5-F | CCGCACCTGCCTCACCATATG |
|  | Ccl5-R | CTTGGCGGTTCCTTCGAGTGAC |
| Ccr2-F | Ccr2-F | CATGCTTACCACAGGCTCATCTCC |
|  | Ccr2-R | TCAGGCAGTCTCCTACCTCATCAG |
| Ido1 | Ido1-F | CAGTGCAGTAGAGCGTCAAGACC |
|  | Ido1-R | GCAGACCTTCTGGCAGCTTGG |
| Ifng | Ifng-F | CAGGCCATCAGCAACAACATAAGC |
|  | Ifng-R | AGCTGGTGGACCACTCGGATG |
| GAPDH | GAPDH-F | GGTGAAGGTCGGTGTGAACG |
|  | GAPDH-R | CTCGCTCCTGGAAGATGGTG |

**Figure S1** Body weight and survival of KO-infected mouse. Data are represented as mean ± SEM. *p < 0.05; **p < 0.01.

**Figure S2** Proportions of CD3^+^CD4^+^ and NK1.1^+^ cells in peripheral blood were evaluated by flow cytometry. Data are presented as mean ± SEM.*p < 0.05; **p < 0.01.
